# The Dynamic Photometric Stereo Method Using a Multi-Tap CMOS Image Sensor †

**DOI:** 10.3390/s18030786

**Published:** 2018-03-05

**Authors:** Takuya Yoda, Hajime Nagahara, Rin-ichiro Taniguchi, Keiichiro Kagawa, Keita Yasutomi, Shoji Kawahito

**Affiliations:** 1 Graduate School of Information Science and Electrical Engineering, Kyushu University, Fukuoka 819-0395, Japan; rin@kyudai.jp; 2 Institute for Datability Science, Osaka University, Osaka 565-0871, Japan; nagahara@ids.osaka-u.ac.jp; 3 Research Institute of Electronics, Shizuoka University, Shizuoka 432-8011, Japan; kagawa@idl.rie.shizuoka.ac.jp (K.K.); kyasu@idl.rie.shizuoka.ac.jp (K.Y.); kawahito@idl.rie.shizuoka.ac.jp (S.K.)

**Keywords:** vision sensor, computational photography, 3D surface recovery, photometric stereo

## Abstract

The photometric stereo method enables estimation of surface normals from images that have been captured using different but known lighting directions. The classical photometric stereo method requires at least three images to determine the normals in a given scene. However, this method cannot be applied to dynamic scenes because it is assumed that the scene remains static while the required images are captured. In this work, we present a dynamic photometric stereo method for estimation of the surface normals in a dynamic scene. We use a multi-tap complementary metal-oxide-semiconductor (CMOS) image sensor to capture the input images required for the proposed photometric stereo method. This image sensor can divide the electrons from the photodiode from a single pixel into the different taps of the exposures and can thus capture multiple images under different lighting conditions with almost identical timing. We implemented a camera lighting system and created a software application to enable estimation of the normal map in real time. We also evaluated the accuracy of the estimated surface normals and demonstrated that our proposed method can estimate the surface normals of dynamic scenes.

## 1. Introduction

3D information acquisition has drawn considerable research attention in recent years and various 3D information acquisition methods have been proposed that use devices such as image sensors and laser scanners. Image sensors have the advantages of small size and low cost and are therefore used for acquisition from scenes in applications such as person identification and factory automation. Many methods are available for 3D surface information acquisition using image sensors. These methods can be separated into two types: active sensing methods and passive sensing methods.

Passive sensing methods such as stereo vision techniques use only image sensors and capture the surfaces of a target scene from different viewpoints. The surface points of the target scene are projected into the corresponding pixels of the captured images along the line of sight and we can then reconstruct the 3D surface based on two or more images and the geometric relationship between the viewpoints. However, the captured images can be affected by backlighting or ambient illumination because they use only standard image sensors. If the lighting conditions change dynamically during capture of multiple images, the appearances of the individual captured images are affected by the lighting, and we cannot then calculate the pixel correspondences among the captured images correctly.

Active sensing methods generally use an image sensor with light sources. The target scene is illuminated using the light sources and the image sensor then captures the light reflectance. For example, light-emitting diode (LED) sources with near infrared and bandpass filters on a camera are usually used to avoid the effects of ambient illumination. Active methods are therefore more robust to environmental lighting changes than passive methods. Many active sensing methods have been proposed, including structured lighting [1], time-of-flight (ToF) [2] and photometric stereo [3] methods.

**Figure 1:**
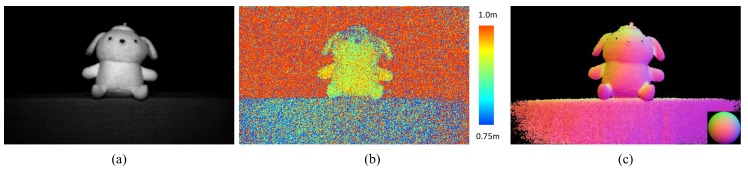
Comparison of time-of-flight (ToF) and photometric stereo methods. (**a**) shows the target scene. (**b**,**c**) show a depth map from the ToF method and a normal map from the photometric stereo method, respectively. The same image sensor was used for both methods. (**b**) shows the absolute depth of the object from the camera. The image noise affected the estimated depth directly and small structures were contaminated by the noise. (**c**) shows an object shape that is smoother and more detailed than that in (**b**).

Structured lighting is an extended method of stereo vision which replaces one of the image sensors to the projector which project the special light patterns directly onto the scene. We can find the correspondences between the projected pattern and captured images, and estimate the depth map by using triangulation. ToF methods use the speed of the light and the phase delay to calculate the scene depth. A ToF camera emits light that is modulated using either sinusoidal or rectangular pulses and receives the light that is reflected from the object. The delay of the light is then calculated by auto-correlation between the emitted light and the reflected light and a depth map is generated as shown in Figure 1b. In contrast, the photometric stereo method [3] uses light emitted from different directions; three or more different lighting and shading images are captured and the normal map is estimated as shown in Figure 1c.

The photometric stereo method estimates the surface normals of the target object while ToF methods estimate the absolute depth of the object from the camera. The photometric stereo method therefore obtains the object shape in greater detail than ToF methods, as illustrated by the comparison in Figure 1. However, the photometric stereo method is based on the following strong assumptions. First, at least three input images acquired under different lighting conditions are required. Second, the camera and the scene should both remain static while all three input images are captured, which means that the pixel intensities of the images are thus only affected by changes in the lighting because each of the pixels in the images should correspond to the same scene points in each image. Under these assumptions, the photometric stereo method provides linear estimates of the normal vector for each pixel based on the intensities of the three images. The classical photometric stereo method therefore cannot be applied to dynamic scenes because the pixels at the same positions in each of the images do not correspond to the same object positions when the scene is dynamic.

However, there is considerable demand for acquisition of detailed object shapes from a dynamic scene in applications such as facial recognition or medical imaging. Gökberk et al. [4] attained the best possible human face recognition performance using the surface normals information as the features of the human face. The photometric stereo method can thus capture better 3D surface features from the human face than ToF methods and the dynamic surface normals estimation process enables analysis of the details of human facial expressions. In an application in the medical field, Vincente et al. used the photometric stereo method for polyp detection [5]. The photometric stereo method was used to obtain the light shapes of a target region that included polyps, and the small polyps were easily recognized from a visualized image of the normal map. However, actual in vivo human tissue is moving and the classical photometric stereo method cannot thus be applied under dynamic conditions. Vincente et al. thus applied the photometric stereo method to *ex vivo* human tissue. A photometric stereo method for use in dynamic scenes could therefore contribute to many more medical applications.

Several approaches have been applied to provide a photometric stereo method that is suitable for dynamic scenes. The solutions can be classified into two main groups based on use of either multi-spectral lighting or high-speed cameras. Christensen et al. [6] and Hernández et al. [7] proposed multi-spectral photometric stereo methods. Their methods used differently colored red, green and blue light sources, with each colored light source being placed at a different position. The captured color images were separated into red-green-blue (RGB) color channels and these separated images were then used to estimate the normals, rather than images that were acquired under different lighting conditions. This method can be used to estimate the normals in a dynamic scene because it needs only a single color image and the different channels of the image are all captured at the same time. Smith et al. [8] used three narrow infrared light sources instead of using RGB color light sources. They used three different bandpass filter and separated the reflectance from each light source. However, these method assumes that the albedos of all objects in the scene are both known and uniform. The classical photometric stereo method generally uses the same light sources for each captured image and assumes that the unknown albedo variable at each pixel is one. If the albedos of the surface are unknown under each colored light source, there are then three unknown albedos at each pixel in the captured images and the problem thus becomes ill-posed. Thus Jankó et al. [9] and Kim et al. [10] tracked each pixel of captured images and calculated surface normals from multiple frames.

Vlasic et al. [11] and Malzbender et al. [12] acquired images for their photometric stereo methods using high-speed cameras with frame rates of 240 fps and 500 fps, respectively. These high-speed cameras can obtain high-speed video sequences and the researchers ignored the differences between the frames of the images, even though the scenes were dynamic. The pixels in the different images are still assumed to correspond and the photometric stereo method can thus be used to estimate the surface normals. However, such high-speed cameras are generally expensive, and the images have lower signal-to-noise ratios (SNRs) because the high-speed camera uses shorter exposure times to produce higher frame rates. These low input image SNRs cause the normal estimation process to be unstable. The photometric stereo method thus requires strong lighting and higher camera sensitivity in this case, as noted by Vlasic et al. [11].

In this paper, we propose a dynamic photometric stereo method based on use of a multi-tap complementary metal-oxide-semiconductor (CMOS) image sensor. The multi-tap CMOS image sensor can divide the electrons from a single photodiode into multiple exposures in a single pixel and can thus obtain multiple images with almost identical timing. We synchronized the light sources with this multi-tap CMOS image sensor to allow the sensor to capture several images under different lighting directions at almost the same time. We built a prototype photometric stereo system consisting of a camera with a multi-tap CMOS image sensor, a lighting system and real-time image capture and processing software. The prototype realized estimation of the normal map at 70.5 fps. We also evaluated the performance of the proposed system and the accuracy of the estimated surface normals. This paper is an extended version of our earlier conference paper on this system [13].

## 2. Photometric Stereo Method

The photometric stereo method was first proposed by Woodham [3]. If an image *I* is captured under illumination by directional lighting $\vec{l}=\left[l_{x}\;l_{y}\;l_{z}\right]$ and the target object has a Lambertian surface, the pixel intensity I(a,b) at (a,b) of the captured image *I* can be given by the following equation:

(1) $$I\left(a,b\right)=\rho \left(a,b\right)\;\vec{l}\;\vec{N}\left(a,b\right),$$

where ρ(a,b) and $\vec{N}\left(a,b\right)$ represent the albedo and the surface normal at pixel (a,b), respectively. Each surface normal $\vec{N}\left(a,b\right)$ is a 1×3 vector of ${\left[n_{x}\;n_{y}\;n_{z}\right]}^{T}$ (T denotes a matrix transpose). The intensity of the captured image is dependent on both the lighting direction and the surface normal vector of the target object. The photometric stereo method can be used to estimate the surface orientation of a target object using three or more images with known lighting conditions. If *i* images were captured under different lighting conditions denoted by $\vec{l^{1}},\vec{l^{2}},\ldots ,\vec{l^{i}}$, then Equation (1) can be rewritten as follows:

(2) $$\left[\begin{array}{c} I^{1}\left(a,b\right)\\ I^{2}\left(a,b\right)\\ \vdots \\ I^{i}\left(a,b\right) \end{array}\right]=\rho \left(a,b\right)\left[\begin{array}{c} \vec{l^{1}}\\ \vec{l^{2}}\\ \vdots \\ \vec{l^{i}} \end{array}\right]\vec{N}\left(a,b\right).$$

We can then rewrite Equation (2) simply as:

(3) $$I=\rho \left(a,b\right)L\vec{N}\left(a,b\right),$$

where I and L represent a 1×i vector and a 3×i matrix, respectively.

We obtained the camera and light source positions prior to calibration, and L was determined from the relative positions of the light sources. The surface normals can therefore be obtained from Equation (5) below. Here, $L^{†}$ represents a pseudo-inverse matrix of the light source positions. The albedo $\vec{\rho }$ and the unit surface normal vector N at (a,b) can therefore be calculated using the following equations:

(4) $$\rho \left(a,b\right)=\left|\begin{array}{c} L^{†}I \end{array}\right|,$$

(5) $$\vec{N}\left(a,b\right)=\frac{1}{\rho (a,b)}L^{†}I.$$

Equation (5) indicates that the photometric stereo method requires at least three images that have been captured under different lighting directions to calculate the surface normals because $L^{†}$ should be a matrix of rank 3. It is also assumed that any intensity change comes from lighting direction changes alone. In addition to this assumption, classical photometric stereo method assumes the target object has Lambertian surface, the light sources are parallel illumination and uniform, the direction of incident illumination is known, there is no cast shadow or occlusions, and the target scene is static. There are numerous research tackling these assumptions, non-Lambertian reflectance [14,15,16,17], surface including specular [18,19,20], cast shadow problem [21], point light source [22,23,24], the non-uniform lighting [25,26,27] and unknown lighting direction [28,29,30]. In case of a point light source, we can rewrite the Equation (2) as follows:

(6) $$I^{i}\left(a,b\right)=\rho \left(a,b\right)\left(\vec{l^{i}}-\vec{x}\right)\vec{N}\left(a,b\right).$$

where $\vec{x}$ is the positon corresponded to the pixel of captured images. To calculate the surface normal exactly, we need to know the coordinates of target surface position. Therefore, many extended photometric stereo methods requires more than three light sources or using optimization to determine the surface normals.

**Figure 2:**
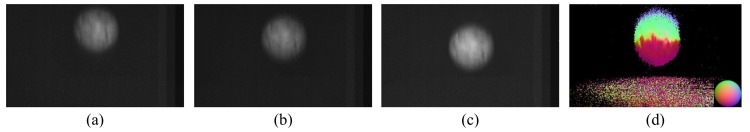
Results of use of classical photometric stereo method for a dynamic scene of a falling ball. (**a**–**c**) represent the three captured images with different lighting directions. The positions of the falling ball do not correspond in the images shown in (**a**–**c**). (**d**) shows the normal map, which is incorrect because the classical photometric stereo method assumes that intensity changes in captured images only come from changes in the lighting conditions. Note that the colors in the estimated image indicate the normal directions of the colored sphere, as shown at the bottom right.

As I mentioned in Section 1, the static scene assumption is one of the important issue to be resolved. In case of a scene is dynamic, in which shapes may change or the object may move, the captured images are not corresponded and would result in the wrong shape being determined, as shown in Figure 2d. The classical photometric stereo method is thus not applicable to dynamic scenes. We propose a method for capturing images almost the ideal timing in spite of the dynamic scene.

## 3. Dynamic Photometric Stereo Method Using Multi-Tap CMOS Image Sensor

### 3.1. Multi-Tap CMOS Image Sensor

CMOS image sensors are becoming increasingly popular in commercial products because they offer system-on-chip integration and low power consumption. Regular CMOS image sensors for photography applications use a single photodiode in each sensor pixel. Each of these photodiodes converts photons into electrons via the photoelectric effect. The electrons are then used to charge a storage diode during the exposure period. The charged electrons are subsequently read out and form a single digital image in which the intensity of each pixel corresponds to the number of electrons and thus to the number of photons.

**Figure 3:**
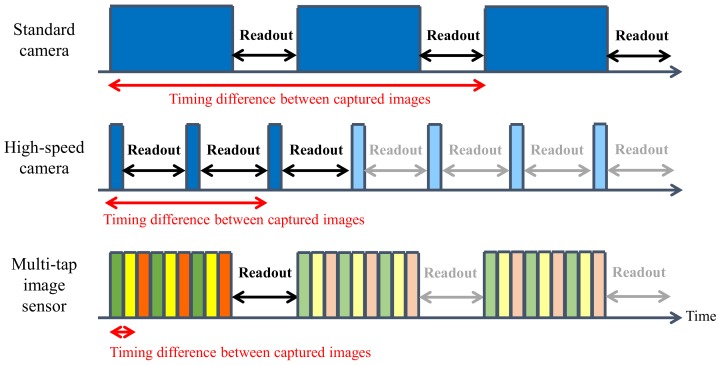
Timing diagram comparison of the exposure and readout times for a standard camera, a high-speed camera, and the multi-tap CMOS image sensor. In the time taken for a standard camera to capture one image, a high-speed camera can capture several images. However, the exposure time of such a camera must be short and the signal-to-noise ratios (SNRs) of the captured images are low. Multi-tap image sensors can acquire almost identical images through iteration of the short exposure time with a high SNR. In this diagram, a three-tap image sensor is used as the multi-tap complementary metal-oxide-semiconductor (CMOS) image sensor.

Figure 3 shows a comparison of the timing diagrams for the exposure and readout times of three different methods. The upper diagram shows a timing diagram for the capture of three images using a standard camera. The rectangle in each diagram represents the image sensor exposure time. As the diagram indicates, the timing differences are dependent on the exposure time and the readout time. Therefore, the timing difference increases when we capture several images using a standard camera. The middle diagram shows the timing diagram for image capture using a high-speed camera. In general, high-speed cameras can capture several images during the time taken to capture a single image using a standard camera. However, the high-speed camera exposure time for each image must be short, and these images thus have low SNRs.

**Figure 4:**
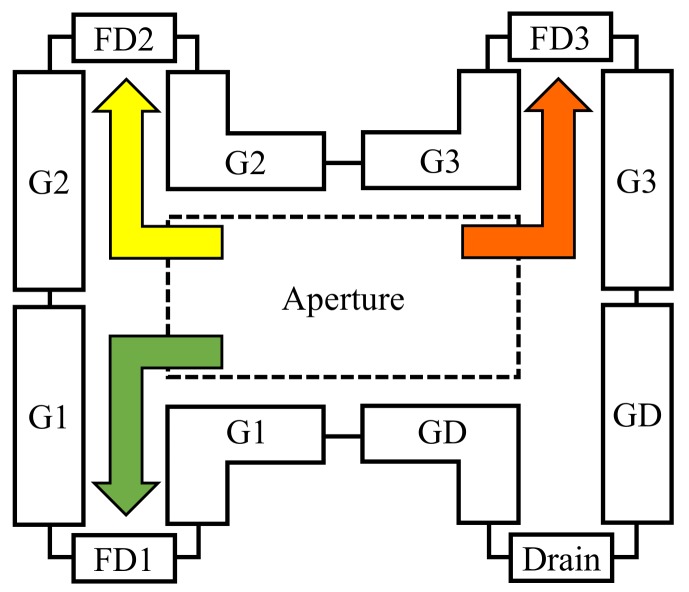
Single pixel structure in a multi-tap CMOS image sensor. The green, yellow and orange arrows represent the electron flows that are generated via the aperture. The colors of the arrows correspond to the colors of the exposures shown in the bottom section of Figure 3. We can select the floating diffusion (FD) in which the electrons are to be stored by changing the gate signals.

The multi-tap CMOS sensor was proposed previously in the literature [31,32]. This sensor contains multiple floating diffusions (FDs) that can split the electrons that are generated by the photodiode of a single pixel to produce multiple exposures and thus form multiple images. Figure 4 shows the structure of a single pixel in the multi-tap sensor [32] that was used in this work. This sensor has an aperture containing a photodiode, three FDs (FD1, FD2 and FD3) and a drain. The pixel also contains four sets of gates (G1, G2, G3 and GD). The multi-tap sensor has a 413 × 240 pixel array, and each pixel shares the gate signals. We can thus select a specific FD and charge the electrons to that FD by changing only four gate signals. When G1 is set to high, the electrons that are generated in the aperture then move to FD1 and are stored there, as indicated by the green arrow shown in Figure 4. When G2 is set to high, the electrons that are generated in the aperture move towards and are stored in FD2, as indicated by the yellow arrow shown in Figure 4. When G3 is set to high, the electrons that are generated in the aperture move towards and are stored in FD3, as indicated by the orange arrow shown in Figure 4. We can thus obtain multiple partitions of the exposures by iterating this process multiple times, as shown in the bottom diagram of Figure 3. The green, yellow and orange colors used for the exposure partitions shown in Figure 3 correspond to the charges of FD1, FD2 and FD3, respectively. We can then obtain three different images through integration of the three different colored regions of the exposures during the readout process at the end of the exposure process. The timing differences among the captured images represent one way of partitioning these exposures. The small exposure time can be short enough to allow the differences among the captured images to be ignored and the multi-tap CMOS image sensor can thus capture several images with almost identical timing. For example, we consider exposure of standard image sensor (upper diagram of Figure 3) is 0.33 ms and small exposure of multi-tap CMOS image sensor (bottom diagram of Figure 3) is 33 μs and the multi-tap CMOS image sensor iterates small exposures 10 times. To obtain the image, we need to readout image one by one in the standard sensor. Therefore, the delay for obtaining three images required for photometric stereo of standard sensor is 0.33 ms × 2 + 13.2 ms × 2 = 27.66 ms. On the other hand, multi-tap exposures alternatively expose the images and the delay between the first image and the third image is only 0.03 ms × 2 = 0.06 ms. Thus SNRs of multi-tap CMOS image sensor is the same to a single tap exposure, however the delay is quite shorter. In addition, multiple iterations of these short exposures allows the multi-tap CMOS image sensor to obtain a sufficient exposure time in total. The multi-tap CMOS image sensor therefore provides a higher SNR when compared with that of high-speed cameras.

This type of multi-tap CMOS image sensor is almost becoming trivial because it is commonly used in depth cameras that use ToF methods, such as the camera in the Microsoft Kinect [33,34] and other similar products [2]. The ToF depth sensor uses light that has been modulated by either sinusoidal or rectangular pulses with nanosecond-scale periods, and we can then calculate the delay of the light by auto-correlation of the emitted light of different intensities among the captured images. However, we require several images to be captured with different nanosecond-scale timings. The multi-tap CMOS image sensor can set changes in the timings of the taps with nanosecond periods and it can also calculate the ToF. We can then calculate the distance between the image sensor and the target scene by simply multiplying the ToF by the speed at which the light is traveling. We apply this multi-tap CMOS sensor to generate a photometric stereo method for use with dynamic scenes in this paper.

### 3.2. Photometric Stereo Method for Dynamic Scene Estimation

**Figure 5:**
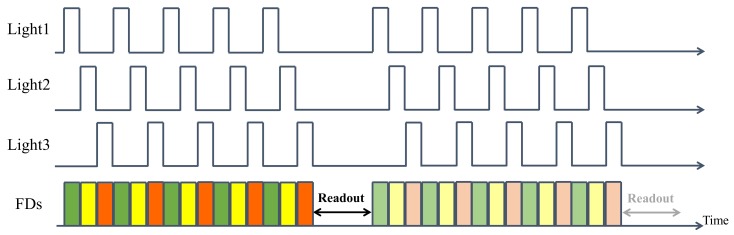
Timing chart for synchronization between the light sources and the exposure times of the different FDs. Each color at the bottom of the figure represents a different FD as shown in Figure 4. The light sources are fully synchronized with the gate signals of each of the FDs. After readout, image1 from FD1 contains only light emitted by light1. Image2 and image3 also only contain light emitted by light2 and light3, respectively.

We aimed to use the multi-tap CMOS image sensor to estimate a normal map for a dynamic scene. The classical photometric stereo method requires the acquisition of at least three images with different lighting directions and thus cannot be applied to dynamic scene estimation. However, the multi-tap CMOS image sensor can capture multiple images with almost identical timing. In our proposed method, the light sources that are required for the photometric stereo method are synchronized with the gate signals of the multi-tap CMOS image sensor. Figure 5 shows the timing chart used for synchronization between these light sources and the exposure times of the multi-tap CMOS image sensor. Each light beam is emitted in the form of multiple iterative pulses and the duration and timing of each light beam is synchronized to a corresponding tap for the exposure. For example, the pulse from light1 is matched to the exposure of FD1. As a result, the image1 obtained from FD1 after the readout process is an image of the target scene that was illuminated by light1 only, while image2 and image3, which were obtained from FD2 and FD3, are the corresponding images when illuminated by light2 and light3, respectively. The light emission periods and the exposure times of the FDs can be reduced to the microsecond scale. This is fast enough to allow the timing differences between the captured images to be ignored, but also allows sufficient time for the scene to be lit separately using several different light sources. We thus obtain the three images with different lighting angles that are required for the photometric stereo method algorithm almost simultaneously. We then apply the standard linear solution [3] for the photometric stereo method to these captured images to realize a dynamic photometric stereo method.

## 4. Implementation

In this section, we describe the implementation of the camera lighting system based on the concept presented in the previous section. To build the camera lighting system, we must first determine the duration of the exposure. There is a trade-off between exposure duration and the noise in the captured images. If the speed of a dynamic scene is high, long exposure times cause motion blur. This effect can be reduced by reducing the exposure time; however, short exposure times cause lower SNRs and this affects the accuracy of the estimated surface normals. We therefore determined the target dynamic scene and designed an appropriate exposure duration. We then created the light sources and built the camera lighting system. We also wrote software to estimate and display the normal map in real time.

We then evaluated the proposed camera lighting system. In the photometric stereo method [3], the light rays are assumed to be parallel. In most cases, the light sources are placed sufficiently far away from the target object to be regarded as parallel light rays. However, the actual light intensity is reduced according to the inverse square law. For example, the light intensity is reduced to one quarter of its original value when the distance doubles, and this causes noise on the captured images. We therefore checked the accuracy of the estimated normal map using the implemented camera lighting system. In addition to the accuracy of the normal map, we verified the intensity correction method for the captured images. The multi-tap CMOS image sensor has different camera sensitivities for each tap, meaning that the captured images are different while the scene and the lighting remain the same. In this paper, we compared two different correction methods, based on use of the same correction ratio over the images and use of different correction ratios at each pixel, to check which of the methods was better for normal map estimation using the multi-tap CMOS image sensor.

### 4.1. Determination of the Exposure Duration

The photometric stereo method can estimate the normal map for a target object from intensity changes in the captured images and the lighting directions. We proposed the use of a multi-tap CMOS image sensor to allow us to capture several images under different lighting conditions almost simultaneously. However, there is a problem that affects the capture of dynamic scenes: motion blur. We need to set an exposure time that is long enough to acquire the detailed intensity changes in the captured images for the photometric stereo method. However, long exposure times cause motion blur of the target object. If the captured images are blurred, then the detailed changes in the image intensity are lost and the normal map will also be blurred. To capture sharp images of dynamic scene, we therefore need to set the exposure time to be of very short duration. However, that also causes the SNR to be reduced and the captured image then becomes noisy. The length of the exposure time is thus very important. The object speed varies and we therefore need to design the suitable exposure durations for specific dynamic scenes.

**Figure 6:**
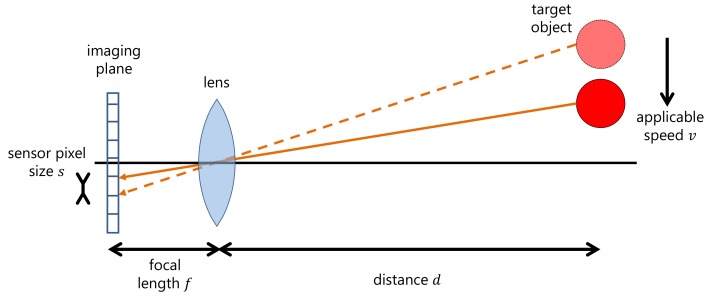
Relationship between object speed and the exposure time (Equation (7)). This figure means that we can ignore the difference of target object position between captured images as long as the target object is projected within the single pixel at imaging plane. This figure shows that the applicable object speed that we can ignore the difference position of moving object depends on a focal length, distance between image sensor and target object, and pixel size of image sensor.

**Figure 7:**
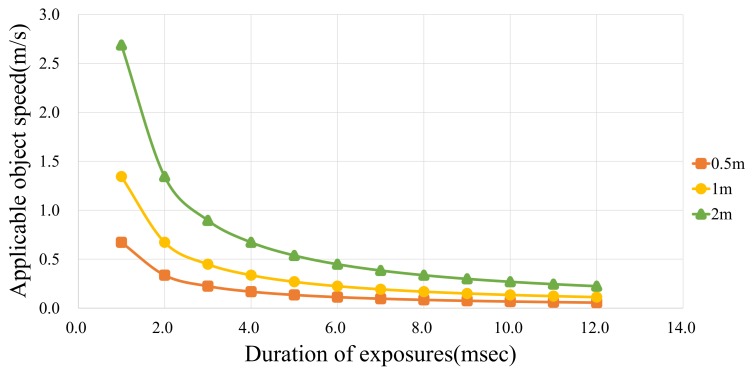
Relationship between applicable object speed and exposure duration for various distances between the camera and the target object (7). In this graph, we calculated the applicable object speed using a sensor pixel size of 16.8 μm and a camera lens focal length of 12.5 mm. As the figure shows, we need to set the exposure duration to be short while placing the target object far away from the image sensor to capture multiple images of a dynamic scene.

The relationship between object speed and the exposure time during which we can obtain the three images required is defined in the following equation:

(7) $$v_{max}=\frac{d}{f}\frac{s}{e},$$

where $v_{max},f$ and *e* are the maximum object speed, the focal length of the camera lens and the total duration of the exposures. *d* represents the distance between the image sensor and the scene, and *s* is the image sensor’s pixel size. This equation means that we can ignore the motion of a target dynamic scene if the target object is projected in the same image sensor pixel over the time as Figure 6. In our implementation, we used the multi-tap CMOS image sensor [32], which has pixel dimensions of 16.8 μm× 16.8 μm. Figure 7 shows the relationship between the applicable object speed and the exposure duration. To obtain the results shown in Figure 7, we calculated the applicable object speed with a camera lens focal length of 12.5 mm. As Figure 7 shows, we can capture several images from a faster dynamic scene by locating the target object further away from the image sensor or reducing the exposure duration.

Next, we consider the optimal exposure setting for a practical example of a dynamic scene. We intend to capture images of a person who is walking at 1.3 m/s at a distance of 1.0 m away from the camera. Equation (7) and Figure 7 show that the optimal exposure time required to avoid motion blur in the image is 1.0 ms.

### 4.2. Camera Lighting System Implementation

Based on the ideal exposure durations, we fabricated three light sources with sufficient lighting power. As we mentioned earlier, the photometric stereo method requires at least three images that have been acquired under different lighting directions. We use the multi-tap CMOS image sensor [32], which can capture three images almost simultaneously. In our case, we need to capture three images with an exposure time of 1.0 ms. We therefore set the exposure times to be 0.33 ms for each image (33 μs × 10 iterations = 0.33 ms including margins). We take a 3 μs margin between each small exposures to make sure the other emitted light don’t come to the exposures. Based on rules of thumbs, we determined the duration of exposure and number of iterations working well in our experimental setup. We used Vishay near infrared LEDs with 870 nm peak wavelength (TSFF5210) for light sources. Each LED have 0.05 W radiant flux, and rise time and fall time is 15 ns. We arranged 16 LEDs into 4 by 4 grid with same pitch. Thus the new three light sources have radiant flux of 0.8 W. We also mounted bandpass filter to image sensor.

**Figure 8:**
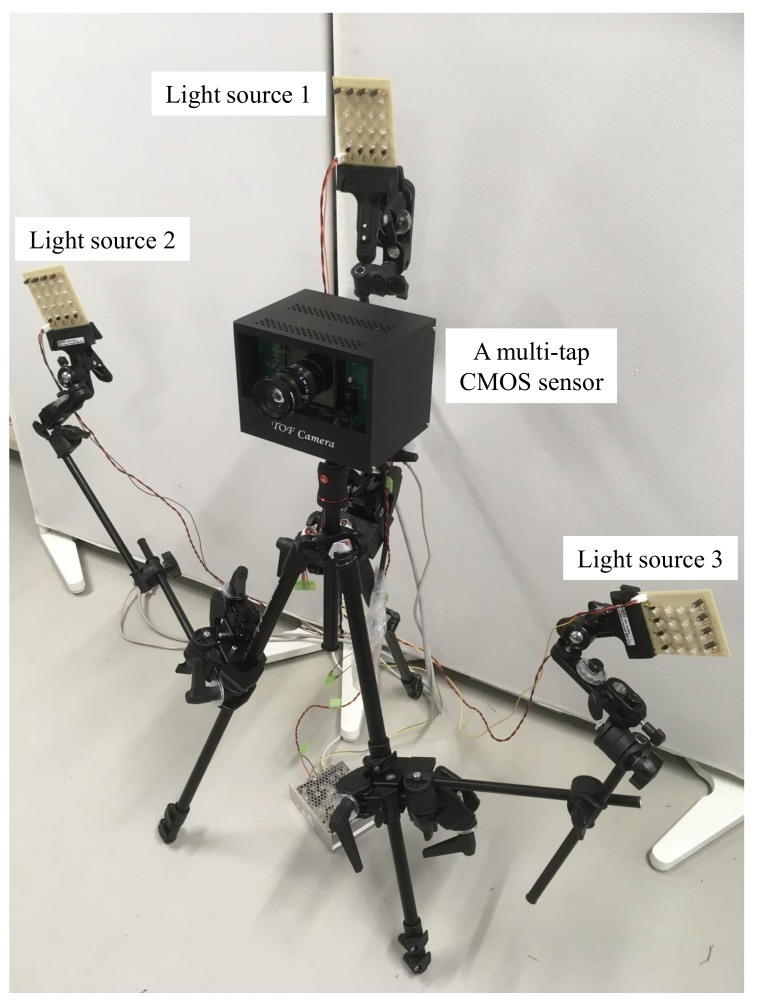
Prototype camera lighting system consisting of multi-tap CMOS image sensor and three light sources. Each light source is composed of 16 LEDs. We arranged the light sources such that the distance between each light source and the target object is equal to reduce the differences due to light attenuation effects. We used an Arduino Uno to synchronize the three light sources with the gate signals of the multi-tap CMOS image sensor.

We implemented the camera lighting system using a tripod. The light sources were mounted on the tripod using three arms. Figure 8 shows a photograph of the camera lighting system. We placed the three light sources around the multi-tap CMOS image sensor, and each light source had same distance from the target scene. The multi-tap CMOS image sensor [32] outputs three gate signals for each FD, and each of the light sources was synchronized with the gate signals. We used an Arduino UNO [35] drives three light sources, which were synchronized by three gate signals as a trigger. The port status change of the Arduino UNO with standard method takes more than 5.0 μs, thus we changed the port register directly and the delay did not occur in our synchronization. Table 1 lists the detailed specifications of our camera lighting system. We wrote the required software application in the C++ language. We used double buffer for capturing images and calculating surface normals. One buffer is used for capturing images and another is used for estimating surface normals. Thus our software can estimate and display the surface normals at 70.5 fps. We used a Windows 7 64-bit personal computer (PC) with an Intel Core i7-5960X central processing unit (CPU; 3.00 GHz) and 8.0 GB of memory.

### 4.3. Normal Map Estimation Error

**Figure 9:**
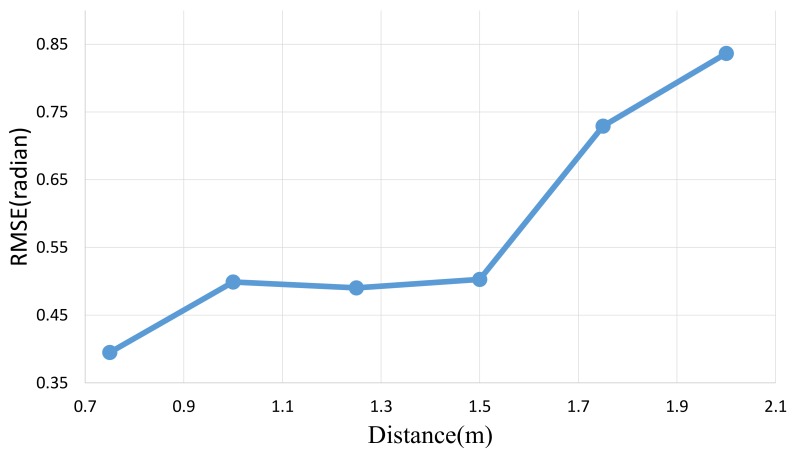
Variation of the accuracy of the normal map with distance from the image sensor. We captured a planar object moving horizontal direction at each distance. The speed of the planar board was 1.3 m/s. We estimated the surface normals using Equation (6) under point light source assumption and calculated the root mean square error (RMSE) in radians between the estimated surface normal and the orthogonal normal vector of the plane using Equation (9). The accuracy decreases as the distance between the image sensor and the target scene increases because of lighting attenuation.

In this section, we evaluate the estimation error of a normal map for different distances between the image sensor and the target scene. As mentioned earlier, the applicable speed of a target scene is dependent on several factors, including distance, exposure duration, the image sensor pixel size, and the focal length of the lens. If the target is located at a greater distance from the sensor, the object motion in the image appears to be slower. There is thus the guarantee that motion blur does not appear if the object is moving at a distance away from the sensor and no error thus comes from motion blur. However, there is another problem: the SNR of the captured images is reduced because of lighting attenuation. The reflected light intensity is attenuated in proportion to the inverse square of the distance from the camera. Therefore, the estimation error increases with increasing object distance. Figure 9 shows the errors of the estimated normal maps at various object distances. We set the exposure time to be 0.33 ms for each image because we assumed that the target object was located 1.0 m from the camera and moving at a speed of 1.3 m/s, as stated in Section 4.1. We used a wooden planar board as a known target object shape. To evaluate the dynamic scene estimation error, the target planar board was moved in horizontal direction by using a robot arm. The moving speed was 1.3 m/s. We used a random dot textured surface board for evaluating the correspondences between captured images. If the captured images of a moving planar board are not corresponded, the estimated surface normals become incorrect. We can assume that a board will have a uniform surface normal (in our experimental setup, that is the camera ray direction). We assumed the perspective projection model, therefore the incidence ray of light is not same at each pixel. We used ${\left[0\;0\;1\right]}^{T}$ vector as the ground truth surface normal. We estimated the surface normals of the planar board by using Equation (6) under point light source assumption, and calculated the errors of surface normals in the form of the root mean square error (RMSE) using the following equation:

(8) $$\theta \left(x,y\right)=\arccos\frac{N_{r}\;\cdot \;N_{c}\left(x,y\right)}{\left|N_{r}\right|\left|N_{c}\left(x,y\right)\right|},$$

(9) $$\mathrm{RMSE}=\sqrt{\frac{1}{wh}\sum_{x=0}^{w}\sum_{y=0}^{h}\theta {\left(x,y\right)}^{2}}.$$

In Equation (8), $N_{r}$ and $N_{c}$ represent the ground truth surface normal of the planar board and the estimated surface normals at coordinates (x,y), respectively. In Equation (9), *w* and *h* represent the width and the height of the captured image, respectively.

As Figure 9 shows, the error increases with increasing distance. This is because the noise caused by lighting attenuation increases and the accuracy of the normal map is affected by this noise. To reduce the noise caused by attenuation of the lighting, the target scene should be closer to the light sources. However, the classical photometric stereo method assumes that the light rays are parallel and the scene remains static. In this experiments, we assumed the light sources were point light sources. As Figure 9 shows, the estimated error becomes small at 0.75 m. This means that the error caused by lighting attenuation is larger than the error caused by motion blur. However we need to set the distance more than 1.0 m from the Equation (7) without motion blur. The error at 1.0 m and 1.5 m are slightly worse than the error at 1.25 m. Finally, the normal map error reaches a minimum when the distance is 1.25 m.

### 4.4. Image Correction Method

We must also consider the sensitivity of a multi-tap CMOS image sensor here. As Han et al. [32] noted, the sensitivity of each tap is different because of imperfect carrier transfer to the FDs. The captured images that are obtained from each tap are therefore different while the scene remains the same. The photometric stereo method assumes that the differences in the intensities of the captured images only come from the different lighting conditions. Most photometric stereo applications use a single tap for exposure. However, we used a multi-tap CMOS image sensor and the different sensitivities of each of the taps may affect the accuracy of the estimated surface normals. We therefore need to correct the sensitivity of the multi-tap CMOS image sensor. Two correction methods are available for images that have been captured using different taps. One method uses the same correction ratio for the entire captured image, as follows:

(10) $$I_{n}^{i}\left(x,y\right)=I_{c}^{i}\left(x,y\right)\frac{\sum^{x,y}I_{b}^{1}\left(x,y\right)}{\sum^{x,y}I_{b}^{i}\left(x,y\right)},$$

where $I_{n}^{i}$, $I_{c}^{i}$ and $I_{b}^{i}$ represent the normalized image, the captured image and the image for use in normalization, respectively. We captured 100 images of a static scene and calculated $I_{b}^{i}$ by averaging these images. In Equation (10), we used the same ratio for the sum of the captured image intensities. However, Equation (10) may not work well when the sensitivity of the taps is different at each pixel. We therefore propose another pixel-wise correction method, as follows:

(11) $$I_{n}^{i}\left(x,y\right)=I_{c}^{i}\left(x,y\right)\frac{I_{b}^{1}\left(x,y\right)}{I_{b}^{i}\left(x,y\right)}.$$

In each correction method, the images $I_{b}^{i}$ must be captured in advance and the whole pixel should be visible.

We compared two correction methods based on the accuracy of their estimated surface normals. In this experiment, we again used a planar board as the target object and we captured a static scene to enable comparison of the correction methods. We set the distance between the multi-tap CMOS image sensor and the target object at 1.25 m, which is the optimal range for surface normal estimation as shown in Section 4.3. We calculated the RMSEs of the surface normals for each correction method and also without correction. Table 2 shows a comparison of the accuracies of the surface normals. As the table shows, the best accuracy was obtained using the pixel-wise correction method (11). This indicates that the sensitivity is different at each pixel in the taps. Additionally, we can correct the intensities of the captured images using Equation (11). In this correction method (11), we use the averaged image for the correction. The captured image actually includes random noise; however, we can assume that the mean of the random noise is zero. We can therefore cancel the effects of the random noise by averaging the captured images and we can correct the sensitivity differences using Equation (11). As a result, we use the pixel-wise calibration method (11) for our camera lighting system.

## 5. Experimental

**Figure 10:**
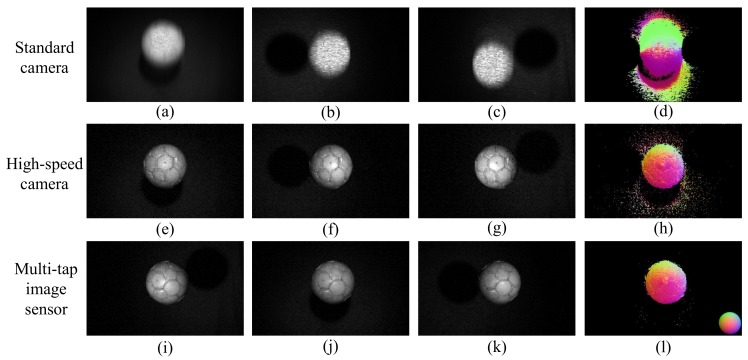
Results of estimation of surface normals of a dynamic scene composed of a falling ball. (**a**–**c**) show images captured with the standard camera settings and (**d**) is the resulting normal map. (**e**–**g**) show images captured using the high-speed camera settings and (**h**) is the resulting normal map. (**i**–**k**) show images captured using the multi-tap CMOS image sensor and (**l**) is the resulting normal map. To make the results easier to see, we modulated the brightness of the captured images to make (**a**–**c**) 300% brighter, (**e**–**g**) 250% brighter and (**i**–**k**) 335% brighter than the original images.

We compared the results estimated using the proposed method with those estimated using the previous photometric stereo method with both a standard camera and a high-speed camera. As the target dynamic scene, we captured images of a falling ball. In the comparison experiments, we set exposure times of 12 ms for each image in the standard camera settings and 0.2 ms for each image in the high-speed camera settings. In the multi-tap CMOS image sensor settings, we set an exposure duration of 30 μs with 10 iterations for each image. The total exposure time for each image is therefore 0.3 ms in the multi-tap CMOS image sensor settings. We used the same image sensor and the same light sources with the different exposure settings listed above for comparison. For the image capture, we set a digital gain of 17.0 dB for the high-speed camera settings and digital gain of 13.0 dB for the multi-tap CMOS image sensor settings. Figure 10 shows a comparison of the captured images and the normal maps for each of these exposure settings.

In the images captured using the standard camera settings (Figure 10a–c), the positions of the falling ball do not correspond and the estimated results of the surface normals (Figure 10d) are incorrect. The position of the falling ball is the same in each of Figure 10e–g,i–k; however the exposure time in the high-speed camera settings was short and this caused some noise. This then affects the estimated surface normals (Figure 10h). In the multi-tap CMOS image sensor settings, the captured image timing is the same and these images have sufficient exposure times for estimation of the surface normals.

**Figure 11:**
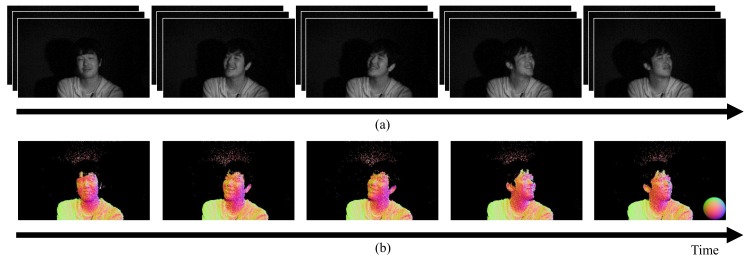
Results obtained using the photometric stereo method for a dynamic scene composed of a facial expression. (**a**) shows the images that were captured using the multi-tap CMOS image sensor, and (**b**) shows the normal maps that were estimated based on these images. We were able to estimate the surface normals of the facial expressions. To make the results easier to see, we modulated the brightness of the captured images to be 800% brighter than the original images.

**Figure 12:**
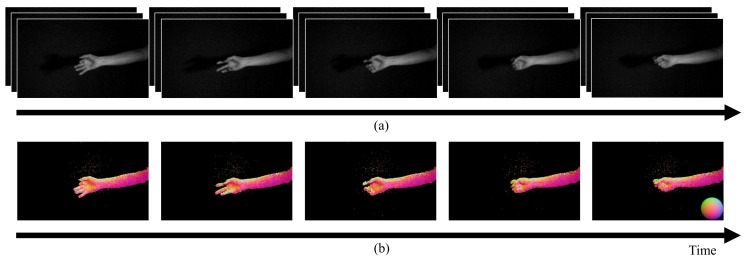
Results obtained using the photometric stereo method for a dynamic scene composed of a hand grasping. (**a**) shows the images that were captured using the multi-tap CMOS image sensor, and (**b**) shows the normal maps that were estimated based on these images. We were able to estimate the surface normals of the deforming object. To make the results easier to see, we modulated the brightness of the captured images to be 800% brighter than the original images.

We applied the proposed method and the related sensitivity correction method (11) to various dynamic scenes, including scenes that contained a moving object and an object during deformation. Figure 11a shows the input images of a facial expression and Figure 11b shows the resulting normal maps with their temporal changes. We can see the surface normals of the human face in each case, despite the fact that the facial expression was changing. Figure 12a shows the input images of a hand grasping and Figure 12b shows the normal map results. The hand shape is changing dynamically in the images. We can see that the surface normals show dynamic changes throughout the time period. We thus confirmed that the proposed method can recover smooth shapes despite both fast object motion and object deformation. We have uploaded movies of these estimated surface normals as Supplementary Material.

## 6. Conclusions

The classical photometric stereo method cannot be used to estimate the surface normals of a dynamic scene because it requires three or more images and corresponding images cannot be acquired using a standard camera. In this paper, we have presented a dynamic photometric stereo method based on use of a multi-tap CMOS image sensor. The multi-tap CMOS sensor can divide a single image sensor exposure and iterate small exposures multiple times. We synchronized the lighting sources with the multi-tap sensor to obtain three images under different lighting conditions but with almost identical timing. We then applied these captured images to the classical photometric stereo method and obtained a normal map of a dynamic scene. We constructed a camera and lighting system to estimate the surface normals of a dynamic scene. In our implementation, we focused on capture of human motion as a target dynamic scene and determined the optimal exposure time and the optimal measurement range. We also created a real-time estimation application that can estimate and display surface normals at 70.5 fps. We performed experiments to confirm that our camera and lighting system can estimate the surface normals of dynamic scenes. The experimental results showed that the proposed camera system could successfully estimate surface normals in actual dynamic scenes.

## Figures and Tables

**Table 1:** Detailed specifications of the implemented camera lighting system.

Properties	Implementation of Camera Lighting System in This Work
Number of pixels	413 (H) × 240 (V) (total number of pixels)
Pixel size	16.8 μm × 16.8 μm
Light source (radiant flux)	**0.8 W** per light source
Exposure time	33 μs × 10 iterations = 0.33 ms for each image
Digital gain	13.0 dB
Readout time	13.2 ms to obtain three images
Frame rate (data stream)	**70.5 fps**
Lens focal length	12.5 mm
Maximum applicable object speed	**1.3 m/s** at a distance of 1.0 m

**Table 2:** Comparison of the captured image correction methods. We set the exposure time to be 33 μs × 10 iterations for each image (including 3 μs margin). We compared the estimated surface normals accuracy with a moving planar board which has a random dot textured surface. The distance between the image sensor and the scene is 1.25 m.

	**No Correction**	**Same Ratio Correction Method over Whole Image** (10)	**Pixel-Wise Correction Method** (11)
RMSE (radians)	0.490	0.433	0.432

## Supplementary Materials

Supplementary video is available online at http://www.mdpi.com/1424-8220/18/3/786/s1.
